# Soybean extract inhibits influenza virus entry: Mechanistic insights

**DOI:** 10.1002/fsn3.4324

**Published:** 2024-08-05

**Authors:** Natsumi Sakata, Yuka Horio, Ryoichi Yamaji, Yuji Isegawa

**Affiliations:** ^1^ Department of Food Sciences and Nutrition, School of Food Sciences and Nutrition Mukogawa Women's University Nishinomiya Hyogo Japan; ^2^ Department of Applied Biological Chemistry, Graduate School of Agriculture Osaka Metropolitan University Sakai Osaka Japan; ^3^ Present address: O‐HARA Gakuen Education Institute Osaka Japan

**Keywords:** clathrin‐dependent endocytosis, influenza virus, soybean, virus entry, virus infection inhibition

## Abstract

Influenza viruses pose significant public health threats because they can cause seasonal outbreaks and global pandemics. Current preventive measures, including vaccines and antiviral drugs, are limited by their low efficacy and the emergence of drug‐resistant viruses. Addressing these issues necessitates the development of novel preventive and treatment methods. Our previous work highlighted the inhibitory effects of soybean hydrothermal extract on influenza virus growth. In this study, we aimed to delve into the mechanism underlying the antiviral activity, specifically the inhibition of viral entry. Our findings reveal that soybean extract significantly inhibited the stages of viral entry during a viral infection and hindered virus uptake by cells. Fluorescence microscopy of stained viral nucleoproteins demonstrated viral localization on the cell membrane in soybean‐treated cells, highlighting a distinctive pattern compared to the control cells where the virus was internalized. Soybean extract targeted the clathrin‐dependent endocytosis pathway, as evidenced by 76% inhibition using a clathrin‐dependent marker (transferrin). The identification of soybean inhibitors underscores the need for further investigation and offers potential for innovative antiviral interventions.

## Graphical abstract

Our study revealed an effect on entry within the antiviral properties of soybean extract. We found that the inhibition of viral entry by soybean extract was an inhibition of clathrin‐dependent endocytosis.

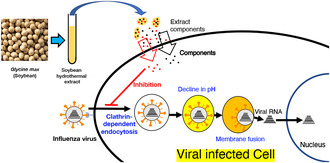

## 1 INTRODUCTION

Influenza viruses pose an annual global public health threat, contributing to significant morbidity and mortality rates, especially among vulnerable populations such as children, the elderly, and those with underlying medical conditions. Serious complications, including pneumonia and encephalopathy, further underscore the gravity of influenza‐related challenges (Grohskopf et al., 2020). Belonging to the Orthomyxoviridae family, influenza viruses are classified into types A, B, C, and D (Hause et al., 2014). Influenza A and B viruses, responsible for human infections and epidemics, present ongoing challenges, with current drugs primarily centering on neuraminidase inhibitors such as zanamivir (Relenza) and oseltamivir (Tamiflu). These drugs hinder viral release during host cell multiplication and are effective primarily in the early infection stages (Kumar, 2011). However, a limitation of their efficacy is exacerbated by the lack of specificity for patients with severe diseases and the emergence of drug‐resistant strains, particularly in children treated with oseltamivir (Kiso et al., 2004). Reportedly, 98% of the HlNl strains isolated during the influenza pandemic period from 2008 to the end of 2009 were oseltamivir‐resistant (Dharan et al., 2009). The development of baloxavir (Zofluza) in 2018 offered an alternative by inhibiting the RNA polymerase of the virus; however, its utility faced challenges with the emergence of drug‐resistant viruses, reported soon after its introduction (Imai et al., 2020).

It is extremely difficult to predict epidemic strains of influenza viruses that mutate rapidly, and the Centers for Disease Control and Prevention (CDC) reported that vaccine efficacy against seasonal influenza from 2004 to 2022 is 10%–60% (Centers for Disease Control and Prevention, 2023). While vaccines against influenza viruses effectively prevent severe illness and complications, they do not impede virus transmission or development. The development of new vaccines, preventive methods, and antiviral drugs with distinct mechanisms is, hence, anticipated to address this.

Over the past decade, various food extracts, including cocoa, onions, and berries, have exhibited anti‐influenza effects (Kamei et al., 2016; Lee et al., 2012; Sekizawa et al., 2013). Recent research underscores the anti‐influenza effect of food‐derived ingredients, particularly tea polyphenols such as catechins, theaflavins, and procyanidins abundant in green tea (Yang et al., 2014). We explored the functionality of *Euglena* as a functional food and zinc as the main antiviral substance (Nakashima et al., 2021). The antiviral activity and major components in Sacna (Seriaceae), namely caffeic acid and luteolin, were investigated by Kanazawa et al. (2022), while the Kalahari watermelon (Cucurbitaceae) exhibited robust antiviral activity and an inhibition mechanism (Hanada et al., 2022; Morimoto et al., 2021). The relationship between polyphenol structures (including modification) and antiviral activity was reported (Morimoto et al., 2022, 2023).

When searching for ingredients in adlay tea that exhibit antiviral effects, soybean, a constituent of the adlay tea we used, showed the strongest activity (Nagai et al., 2018). Daidzein has been identified as an antiviral component found in large amounts in soybeans (Nagai et al., 2019), and detailed aspects of its mechanism of action and signal transduction (Horio et al., 2020, 2023) have been studied. However, daidzein primarily inhibits the viral growth phase, not the attachment or entry phases (Horio et al., 2020). Influenza virus enters cells through clathrin‐dependent and non‐clathrin‐dependent routes (Sieczkarski & Whittaker, 2002). The clathrin‐dependent route is inhibited by monodancylcadaverin (MDC; Ikari et al., 2015). Bafilomycin is an inhibitor of V‐ATPase and inhibits endosomal acidification (Mauvezin et al., 2015), and both inhibitors suppress the nuclear entry of influenza virus genes. Methyl‐β‐cyclodextrin (MβCD) inhibits caveolae‐dependent endocytosis (Sieczkarski & Whittaker, 2002). Although an isoflavone of genistein found in large amounts in soybeans, such as MβCD, inhibits caveolae‐mediated endocytosis, genistein does not inhibit influenza virus entry (Sieczkarski & Whittaker, 2002). These varied viral inhibition mechanisms, acting directly on the virus or indirectly on host cells to suppress the virus, underscore the importance of elucidating viral infection inhibition mechanisms.

Consequently, we aimed to scrutinize the mechanism of influenza virus inhibition by hot water soybean extracts and identify targeted inhibition reactions to search for components in soybeans.

## 2 MATERIALS AND METHODS

### 2.1 Cell lines and viruses

Madin–Darby canine kidney (MDCK) cells, used for the influenza virus growth and virus‐inhibitor assay, were cultured in Minimum Essential Medium (MEM: FUJIFILM Wako Pure Chemical Corporation, Osaka, Japan), supplemented with 7% Fetal Bovine Serum (FBS; Biowest Biotechnology Company, France). CV‐1 and HeLa cells, used for cell‐fusion assay via virus and virus‐endocytosis assay, respectively, were grown in Dulbecco's Modified Eagle's medium (DMEM: FUJIFILM Wako), supplemented with 10% FBS.

Influenza A virus H1N1 (PR/8/34) was used as the strain. The virus culture was performed according to the method described by Morimoto et al. (2022).

### 2.2 Preparation of soybean extracts and viral yield determination in the presence of soybean extracts

Yellow soybeans (variety: Sachiyutaka, harvested in 2018) were obtained from Tottori Prefecture, Japan. The soybeans underwent roasting (180°C, 240 min), grinding with a mixer, followed by hot water extraction (80°C, 90 min), achieved by adding 50 mL of ultrapure water to 5 g of soybeans. After hot‐water extraction, the samples were filtered, lyophilized, and then reconstituted in ultrapure water. Sterilization was performed using a 0.45 μm filter, and the samples were stored frozen at −30°C until use.

The cells were cultured as monolayers in 24‐well flat‐bottomed plates (Thermo Fisher Scientific, Waltham, MA, USA). MDCK cells were washed twice with serum‐free MEM, and the viral stock was diluted to an MOI of 0.001 in MEM containing 0.04% BSA. Following infection for 1 h in a CO_2_ incubator, cells were washed once with serum‐free MEM and incubated for 24 h in DMEM containing 0.4% BSA and 2 μg/mL acetyltrypsin at a volume of 500 μL/well of the adjusted sample, and only the supernatant was collected.

For HeLa cells, similar procedures were followed, with the MOI set at 1 and the incubation period extended to 48 h. Supernatants were collected for subsequent evaluation of virus titers by the focus‐forming reduction assay (FFRA) and the indirect peroxidase staining method, as described in the sections below.

### 2.3 FFRA of viral activity

Viral titers were measured using the method described by Morimoto et al. (2021). Virus‐infected MDCK cells in 96‐well flat‐bottom plates (Corning Inc., Corning, NY, USA) were subjected to indirect peroxidase staining to determine viral titers. Focus staining involved the sequential addition of murine monoclonal anti‐HA antibody (C179 for A (H1N1) viruses, Okuno et al., 1993) and a goat anti‐mouse IgG antibody conjugated to horseradish peroxidase (AP124P; Merck KGaA, Darmstadt, Germany). The development of the peroxidase reaction was according to a procedure described by Graham and Karnovsky (1966). The number of foci in the immunostained infected cells was determined using an inverted light microscope.

### 2.4 Cell viability determination

Cell viability was assessed using the Cell Proliferation Kit I (MTT) (F. Hoffmann‐La Roche Ltd., Basel, Switzerland) based on the method described by Morimoto et al. (2021).

### 2.5 Time‐of‐addition assay

The time‐of‐addition assay was performed using a modified version of a previously described procedure (Nagai et al., 2018). As described above, MDCK cells were plated in 24‐well plates, rinsed twice with serum‐free MEM, and inoculated with A/PR/8/34 (MOI = 0.01). Subsequently, the cells were rinsed twice with serum‐free MEM and incubated in DMEM for 1 h, as described above. Serum‐free DMEM (500 μL/well) containing 1 mg/mL soybean extract, equivalent to approximately seven times its median inhibitory concentration (IC_50_), was introduced at different time points: between 1 h before infection and the time of infection (−1 to 0 h, corresponding to adsorption and entry), and between 0 and 4 h, 4–8 h, or 0–8 h after infection (replication). After each incubation period, the cell monolayers were rinsed twice with serum‐free MEM, and the medium was replaced with a fresh medium. The cells were then cultured for 8 h post‐infection. Subsequently, to collect all virus particles inside infected cells and those bound to the cells, the infected cells were frozen at −80°C and subjected to two freeze–thaw cycles before determining viral yields through the focus‐forming assay.

### 2.6 Viral adsorption inhibition assay

MDCK cells were cultured in a monolayer within 12‐well flat‐bottomed plates (Thermo Fisher Scientific) and washed twice with serum‐free MEM. Subsequently, the virus solution was diluted to 10 MOI in 0.04% BSA‐containing serum‐free MEM. The prepared sample was added, allowing the cells to adsorb the virus on ice or in an incubator for 1 h. Following two PBS washes, RNA extraction was performed using NucleoSpin RNA (MACHEREY NAGEL) according to the manufacturer's instructions. Subsequently, cDNA synthesis was carried out using ReverTrace (TOYOBO). Primers were designed for viral HA of the PR8 virus and the housekeeping gene GAPDH for normalization, and real‐time PCR was performed with a THUNDER BIRD SYBR mixture (TOYOBO), following the protocol described by Choi et al. (2017). The primer sequences used in this study are listed in Table 1.

**TABLE 1:** Primer sequences used in this study for quantitative real‐time PCR analysis.

Target	Primer name	Sequence (5′ to 3′)
HA mRNA^a^	HA‐For	TTGCTAAAACCCGGAGACAC
	HA‐Rev	CCTGACGTATTTGGGCACT
GAPDH mRNA^b^	GAPDH‐For	ACCACCGTCCATGCCATCAC
	GAPDH‐Rev	GTGAGCTTCCCGTTCAGCTC

^a^ HA: influenza virus H1N1 (A/PR/8/34) gene.

^b^ GAPDH: housekeeping gene.

### 2.7 Cell‐fusion inhibition test

Cell–cell fusion was accomplished by a slightly modified method described by Okuno et al. (1993). CV‐1 cells were cultured in a monolayer on 24‐well flat‐bottom plates (Thermo Fisher Scientific) and washed twice with serum‐free MEM. Following infection with a viral stock diluted to an MOI of 0.01 in serum‐free MEM containing 0.04% BSA for 1 h in a CO_2_ incubator, cells were washed once with serum‐free MEM. Subsequently, serum‐free DMEM containing 0.4% BSA and 2 μg/mL acetyltrypsin was added at 500 μL/well and incubated in a 37°C CO_2_ incubator for 24 h. After two washes with serum‐free DMEM, 10 μg/mL serum‐free DMEM containing acetyl trypsin was added (500 μL/well) and incubated in a CO_2_ incubator for 20 min. Following two washes with serum‐free DMEM, cells were incubated with the prepared samples in serum‐free DMEM for 30 min.

Further treatment with serum‐free RPMI (pH 5.0) containing 2% BSA and 10 mmol/L citric acid‐Na_2_HPO_4_ buffer for 2 min preceded two washes with serum‐free DMEM. Serum‐free DMEM containing 2% FBS was added (500 μL/well), and the cells were incubated in a CO_2_ incubator for 3 h. The cells were then fixed with methanol, stained with Giemsa stain, washed with distilled water, dried thoroughly, and observed under a microscope. The fusion index (Isegawa & Okuno, 2018) was calculated as follows:

$$\text{Fusion index}=1-\left(\frac{\text{number of cells}}{\text{number of nuclei}}\right)$$

### 2.8 Observation of pH in endosomes/lysosomes (acridine orange staining)

The impact on cellular endosomal and lysosomal acidification was examined through biological staining with acridine orange (AO) (Garozzo et al., 2011). MDCK cells were cultured as monolayers in 24‐well flat‐bottom plates (Thermo Fisher Scientific) and rinsed twice with MEM‐medium. Soybean hot‐water extract (IC_80_ = 0.35 mg/mL) and 100 nM bafilomycin A1 (Funakoshi: V‐ATPase inhibitor) were added and incubated in a CO_2_ incubator for 1 h. AO, prepared in PBS at a final concentration of 2.5 μg/mL, was added and stained at 37°C for 10 min in the dark. The cells were washed four times with PBS, exposed to a PBS solution containing 0.04% BSA (2 × MEM:PBS = 1:1), and observed under a fluorescence microscope with a BZ‐X filter GFP (OP‐87763) (KEYENCE BZ‐X800; KEYENCE, Osaka, Japan).

### 2.9 Influenza virus uptake analysis

The cells were cultured on a cover glass (12 mm diameter; Paul Marienfeld GmbH & Co. KG) and washed twice with serum‐free MEM. MDCK cells were then exposed to a 10 MOI in 0.04% BSA‐containing serum‐free MEM containing the sample, while HeLa cells were exposed to a 0.04% BSA‐containing serum‐free DMEM virus solution. The cells were incubated at 37°C in CO_2_ for 1 h. Following the removal of non‐bound virus from the cells with serum‐free MEM, 0.4% BSA and serum‐free DMEM containing 2 μg/mL acetyl trypsin, including the sample, were added and incubated for an additional 1 h. After incubation, the cells were washed with serum‐free MEM and 1× PBS, fixed with 3.7% paraformaldehyde for 30 min, and washed again with 1× PBS. Subsequently, acetone‐methanol (−20°C) was applied for 5 min, and cells were dried. Following permeabilization with 1× PBS containing 0.25% Triton X‐100, cells were blocked with PBS containing 3% BSA for 30 min. Staining was performed using a mouse monoclonal Anti‐Influenza A Virus Nucleoprotein antibody [C43] (500 × dilution) as the primary antibody and Alexa Fluor 594 labeled goat anti‐mouse IgG (Invitrogen) (1000‐fold dilution) as the secondary antibody. The cells were allowed to react for 1 h at room temperature (approximately 25°C). Subsequently, the cell nuclei were stained with DAPI (Dojindo, Kumamoto, Japan), and the cells were mounted on glass slides (Matsunami Glass Co., Ltd.) with an inclusion agent (Nippon Genetics), covered with a cover glass, and sealed with nail polish. The cells were then observed under a fluorescence microscope with a BZ‐X filter Texas Red (OP‐87765) for Alexa Fluor 594 and a BZ‐X filter DAPI (OP‐87762) for DAPI (KEYENCE BZ‐X800).

### 2.10 Transferrin uptake analysis

Cellular uptake of transferrin, internalized via the clathrin‐dependent endocytic pathway (Mettlen et al., 2010; Ramachandran, 2011), was observed through fluorescence microscopy. Monodansyl cadaverine (MDC: 150 μM) was used as an inhibitor of clathrin‐dependent endocytosis. The HeLa cells were cultured on a cover glass and washed twice with serum‐free DMEM. Subsequently, the cells were incubated in a 37°C, CO_2_ incubator for 10 min to facilitate the incorporation of transferrin into the cells. After incubation, the cells were washed with 1× phosphate‐buffered saline (PBS) and fixed with 3.7% paraformaldehyde for 30 min. Cell nuclei were stained with DAPI (1000× dilution). A cover glass was placed on a glass slide using an inclusion agent and sealed with nail polish. The cells were then examined under a fluorescence microscope with a BZ‐X filter TexasRed and a BZ‐X filter DAPI (KEYENCE BZ‐X800).

Fluorescence intensity was measured by analyzing fluorescence photographs using ImageJ (version 1.53 g, 4 December 2020: https://imagej.nih.gov/ij/). The region of interest (ROI) was defined based on the cell morphology, and the average fluorescence intensity of the ROIs from 30 cells was calculated.

### 2.11 Uptake analysis of cholera toxin B (CTxB)

We examined the caveolae‐dependent endocytic pathway using CTxB (Parton & Richards, 2003) along with the effect of hydrothermal soybean extract on caveolae‐dependent endocytosis, a process facilitated by the cholesterol‐binding protein, caveolin, which orchestrates vesicle formation for substance uptake (Parton & del Pozo, 2013). Methyl‐β‐cyclodextrin (MβDC: 6 mM) was used as an inhibitor of caveolae‐dependent endocytosis. The HeLa cells were cultured on a cover glass and washed twice with serum‐free DMEM. After a 30 min incubation of the cells in a CO_2_ incubator at 37°C with DMEM containing 0.5% BSA and the sample, 1 μg/mL Alexa Fluor 488 bound CTxB (Invitrogen, C‐34775) was added to the medium and cells. Incubation proceeded for 5 min at 37°C in a CO_2_ incubator. Subsequently, cells were washed with 1× PBS and fixed with 3.7% paraformaldehyde for 30 min. Cell nuclei were stained with DAPI (1000× dilution). The cover glass, mounted on a glass slide with an inclusion agent, was sealed with nail polish. The observation was under a fluorescence microscope with a BZ‐X filter GFP for Alexa Fluor 488 and a BZ‐X filter DAPI for DAPI (KEYENCE BZ‐X800). Fluorescence intensity measurement followed the procedure outlined in Section 2.10.

### 2.12 Statistical analyses

The amounts of virus at the time of addition and during antiviral assays were analyzed using a Student's *t*‐test (two‐tailed) in Excel Toukei (version 6.0; Esumi, Tokyo, Japan). Values are presented as means ± standard deviation (SD). A *p*‐value < .05 was considered significant.

## 3 RESULTS

### 3.1 Anti‐influenza virus activity of soy hot water extract

**FIGURE 1:**
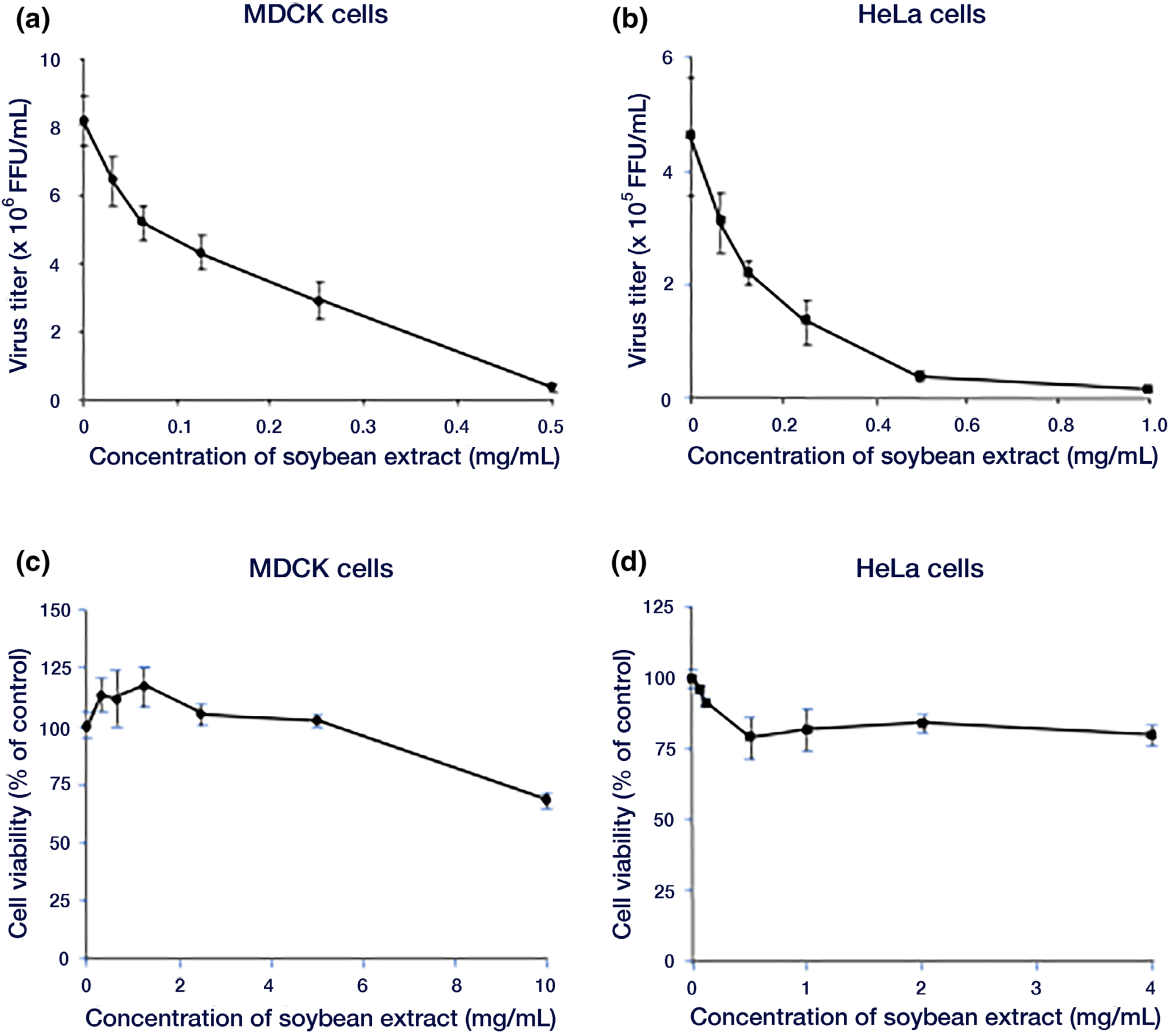
Effect of hot water soybean extract on influenza viral multiplication in MDCK cells. (a, b) Inhibitory effect of soybean hydrothermal extract on virus growth. (a) MDCK cells were infected with 0.001 MOI of the influenza A/PR/8/34 virus for 1 h. Virus titers in culture supernatants 24 h after infection and the addition of sample extracts were determined by FFRA. (b) HeLa cells were infected with 1 MOI of influenza A/PR/8/34 virus for 1 h. After infection, sample extracts were added, and viral titers in the culture supernatant 48 h later were measured by FFRA. (c, d) Cytotoxicity of soybean hot water extract, measured by MTT assay. (c) MDCK cells. (d) HeLa cells. Data are presented as mean ± standard deviation (*n* = 3). The data are representative of three independent experiments. FFRA, Focus‐forming reduction assay; MDCK, Madin–Darby canine kidney; MOI, Multiplicity of infection.

Virus titers in the culture supernatants of the samples and sample‐naïve groups were measured. As shown in Figure 1a,b, the viral titer exhibited a concentration‐dependent reduction following the addition of the sample, confirming the anti‐influenza virus activity of the hot‐water soybean extract against the H1N1 A/PR/8/34 strain. The concentration for 50% inhibition of virus replication (IC50) was determined to be 0.142 ± 0.01 mg/mL for MDCK cells and 0.123 ± 0.01 mg/mL for HeLa cells. No cytotoxicity was observed at the concentrations used in the experiments (Figure 1c,d).

### 3.2 Examination of the stage of inhibition of virus proliferation

**FIGURE 2:**
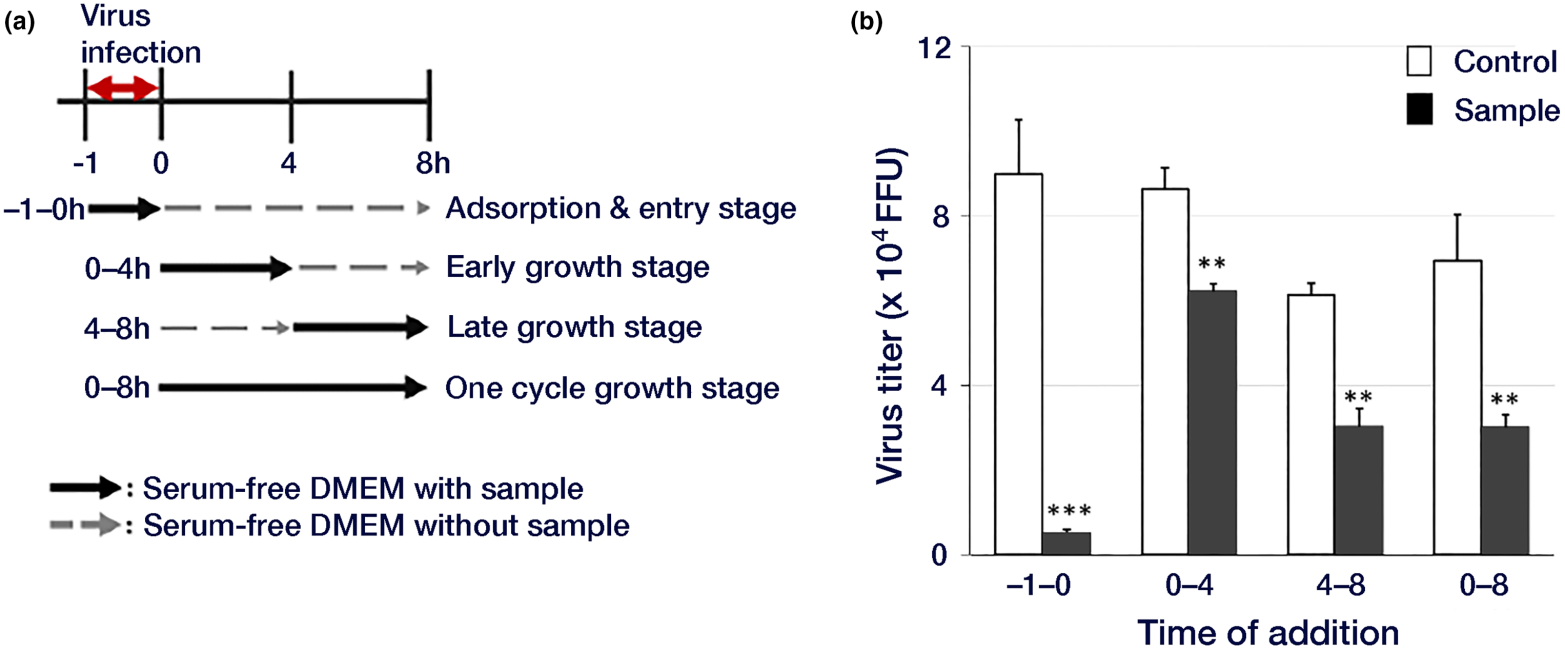
Viral growth inhibition by soy hot water extract. MDCK cells were infected with 0.01 MOI of the influenza A/PR/8/34 virus for 1 h. Serum‐free DMEM (500 μL/well) with or without (control) 1 mg/mL soybean extract was exchanged at different time points. (a) Addition assay schedule. (b) FFRA assay results are indicated by open columns representing the viral yields of control cells and closed columns representing the viral yields of cells treated with soybean extract. Data are presented as mean ± standard deviation (*n* = 3). The data are representative of three independent experiments (**p < .01, ***p < .001). DMEM: Dulbecco's Modified Eagle's medium.

Considering that the growth cycle of the influenza virus in MDCK cells spans 8 h per cycle, we investigated the stage at which soybean hydrothermal extract inhibits virus proliferation by sequential introduction into infected cells. The virus titer was measured, revealing a reduction at all stages, including the adsorption and entry stage, early culture stage, late culture stage, and across all culture stages. Particularly robust inhibition was noted during the −1 to 0 h period, corresponding to the time of viral infection (Figure 2).

### 3.3 Inhibitory effect of soybean hydrothermal extract on influenza virus adsorption

**FIGURE 3:**
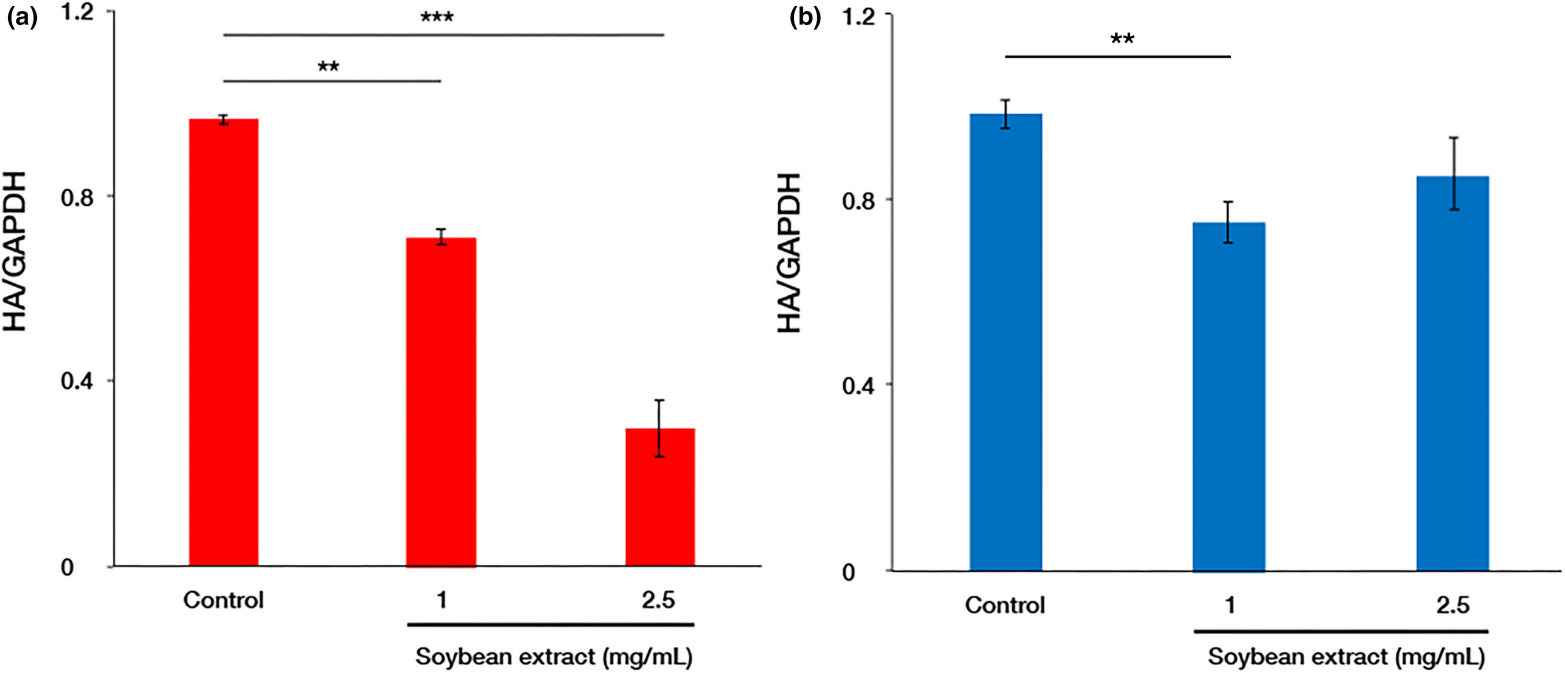
Viral adsorption inhibition by soybean hot water extract. Influenza A/PR/8/34 virus was added at 10 MOI to MDCK cells and incubated in an incubator (a) or on ice (b) for 1 h, followed by RNA extraction and reverse transcription to synthesize cDNA. The total RNA expression of the viral HA protein adsorbed onto cells was measured by qPCR using a 7500 Fast Real‐time PCR System (Life Technologies Japan Ltd.). (a) Red columns indicate the viral adsorption onto cells at 37°C at three different concentrations of soybean extract. (b) Blue columns indicate the same at 4°C. Data are presented as mean ± standard deviation (*n* = 3). The data are representative of three independent experiments (**p < .01, ***p < .001).

Owing to the robust inhibition of the initial stage of viral infection by the hydrothermal soybean extract, we initially examined its impact on the adsorption of viruses to cell membranes. After a 1‐h viral infection period, both viruses internalized into cells, and those adsorbed onto cell membranes were quantified using real‐time PCR, revealing a concentration‐dependent inhibition of viruses upon the addition of the sample at 37°C (Figure 3a). It inhibited energy‐dependent entry. Under low‐temperature conditions (4°C), where the virus remained on the cell membrane, adding 1 mg/mL of soybean extract reduced the virus. However, there was no concentration dependence of the additive on adsorption inhibition (Figure 3b). These observations implied that the hot‐water soybean extract did not influence virus adsorption to cells but impeded energy‐dependent entry.

### 3.4 Inhibition of membrane fusion by hydrothermal soybean extract

**FIGURE 4:**
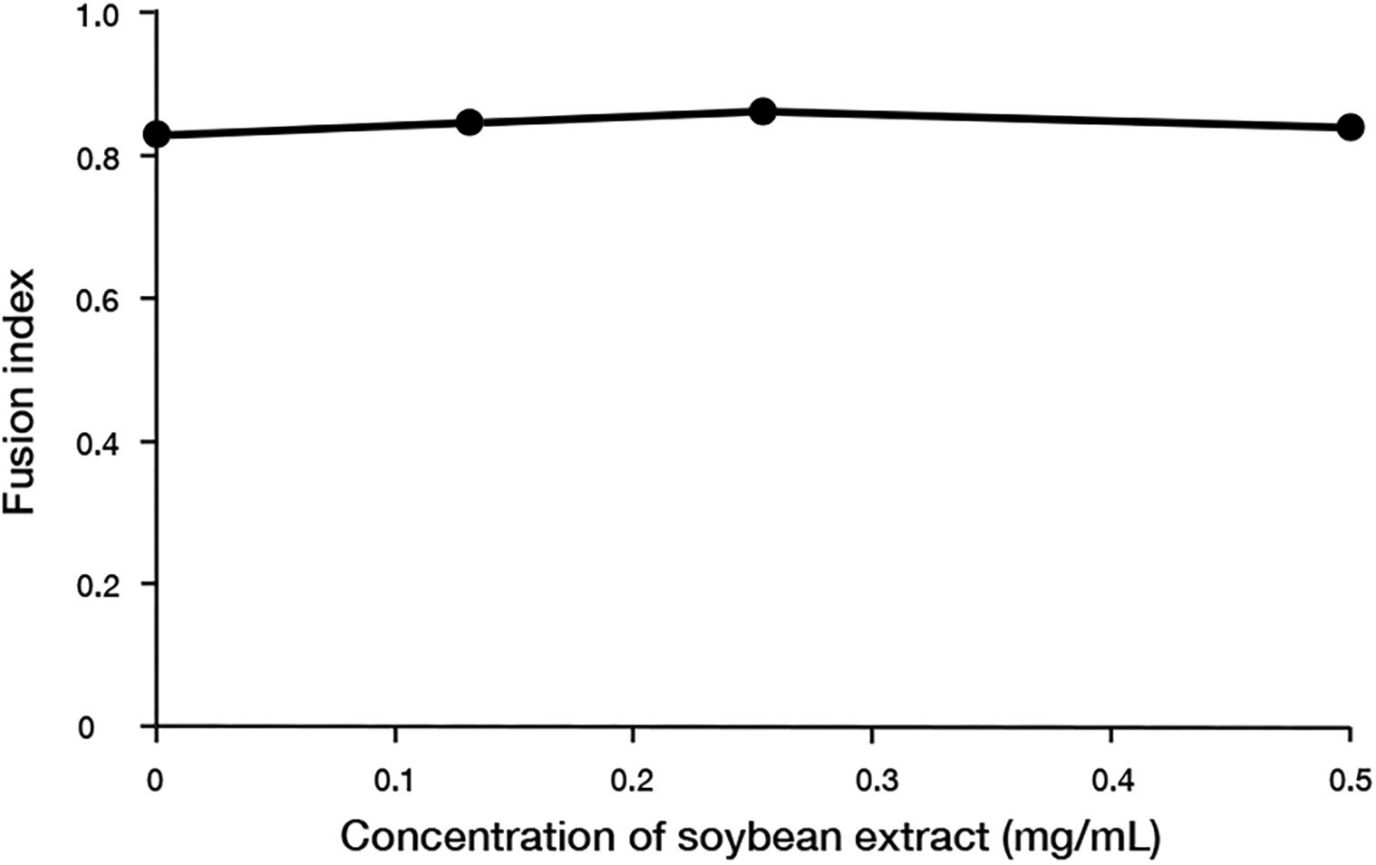
Membrane fusion inhibition by soybean hot water extract. CV‐1 cells were infected with the influenza A/PR/8/34 virus at 0.001 MOI for 1 h and cultured for 24 h post‐infection. Sample extracts were added for 30 min, followed by low‐pH treatment for 2 min and a 3‐h incubation. Subsequently, cells were fixed, stained with Giemsa stain, and observed under a microscope. The data are presented as mean ± standard deviation (*n* = 3) and represent three independent experiments. Fusion index = 1 − (number of cells/number of nuclei).

Since the hydrothermal soybean extract demonstrated no effect on virus adsorption, we investigated its effect on the membrane fusion process between the virus and the cell membrane. To investigate the effect of hydrothermal soybean extract on membrane fusion, we experimented to calculate the fusion index of multinucleated giant cells generated by virus‐induced membrane fusion under acidic medium (pH 5.0) conditions (Figure 4). The results revealed a consistent fusion index in the presence of the sample, indicating no discernible alteration.

### 3.5 Impact of hydrothermal soybean extract on endosomal‐lysosomal acidification

**FIGURE 5:**
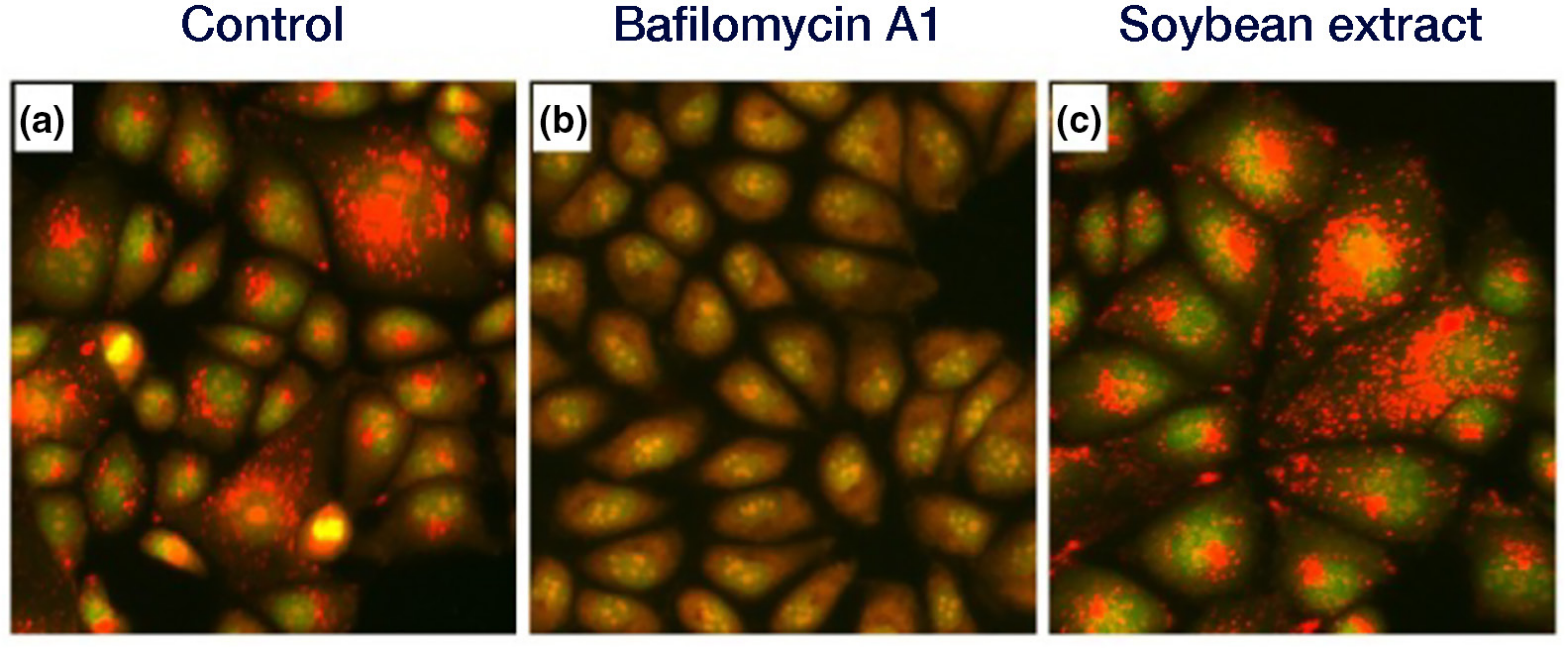
Acridine orange (AO) staining. Each sample was added to MDCK cells and incubated at 37°C for 1 h. After incubation, AO (2.5 μg/mL) was added, and the cells were stained for 10 min. Subsequently, the cells were observed under a fluorescence microscope. (a) Control, (b) 100 nM bafilomycin A1 (V‐ATPase inhibitor), (c) Soybean hot water extract (0.35 mg/mL).

Confirmation of the hydrothermal soybean extract's effect on endosomal‐lysosomal acidification, a key factor in membrane fusion, was by staining with acridine orange (AO), which emits red and orange fluorescence signals under low pH conditions. Control cells exhibited red and orange fluorescence, whereas cells treated with bafilomycin A1 (a V‐ATPase inhibitor), known to inhibit endosomal lysosomal pH reduction, displayed an absence of red and orange fluorescence (Figure 5). Notably, cells treated with the hydrothermal soybean extract displayed red and orange fluorescence akin to that of the control cells. This affirmation established that the hydrothermal soybean extract did not inhibit the acidification of endosomes and lysosomes.

### 3.6 Effect of hydrothermal soybean extract on virus uptake

**FIGURE 6:**
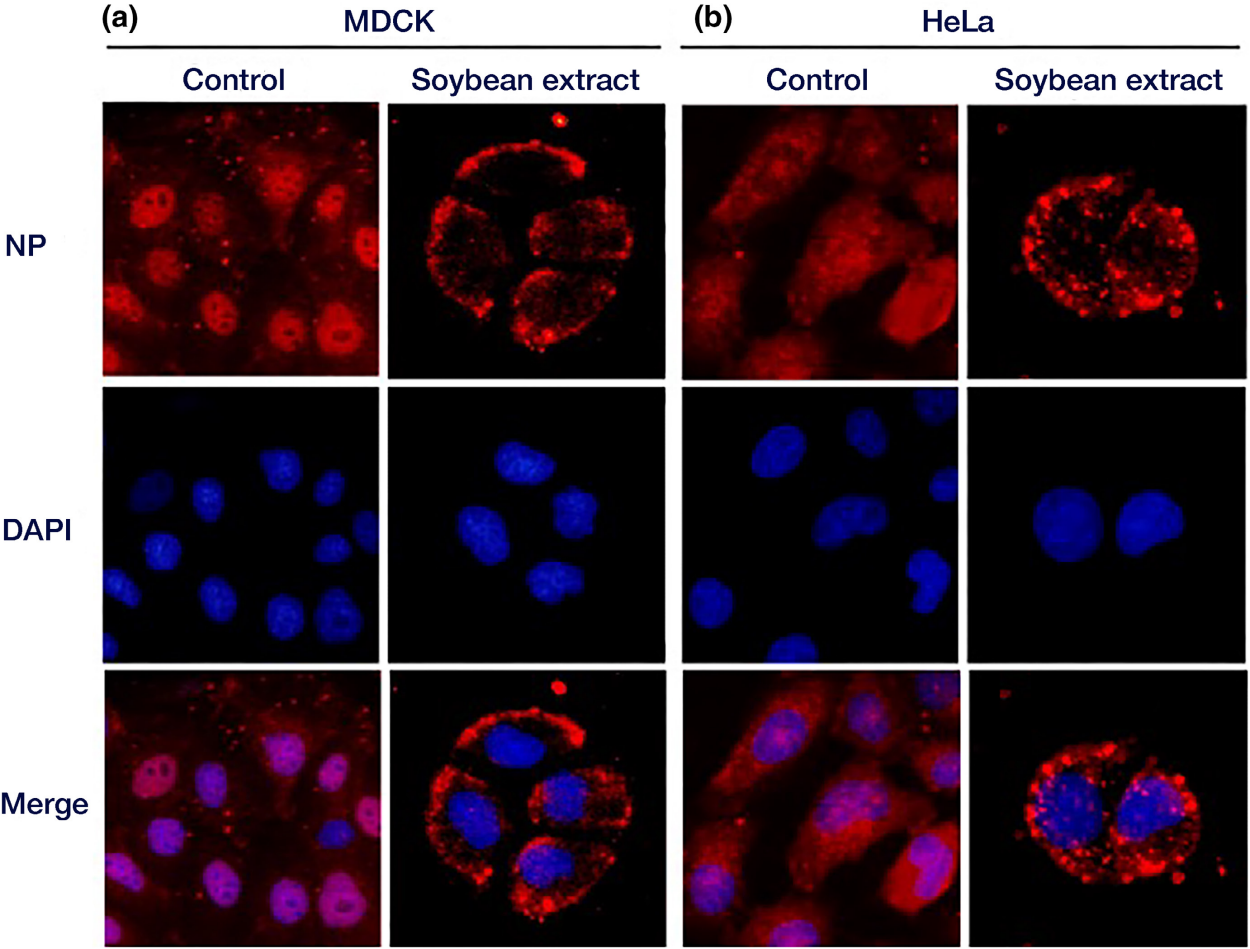
Localization of influenza virus NPs. MDCK and HeLa cells were infected with 10 MOI of influenza A/PR/8/34 virus for 1 h. The cells were fixed after incubation for 1 h. Fluorescence microscopy was used to observe the cells. (a) MDCK cells. (b) HeLa cells. Red: Influenza virus NPs; blue: Cell nuclei.

The impact of the hydrothermal soybean extract on viral uptake into cells was examined by staining and observing the influenza virus NPs. Fluorescence microscopy of cells cultured for 1 h after infection showed that the virus was localized on the plasma membrane of cells treated with hydrothermal soy extract. In contrast, control cells exhibited staining in the cytoplasm and nuclei, indicating the incorporation of the virus into the cells. Based on this observation, we inferred that the hydrothermal soybean extract inhibited viral entry into the cell and endocytosis (Figure 6).

### 3.7 Effect of hydrothermal soybean extract on transferrin uptake

**FIGURE 7:**
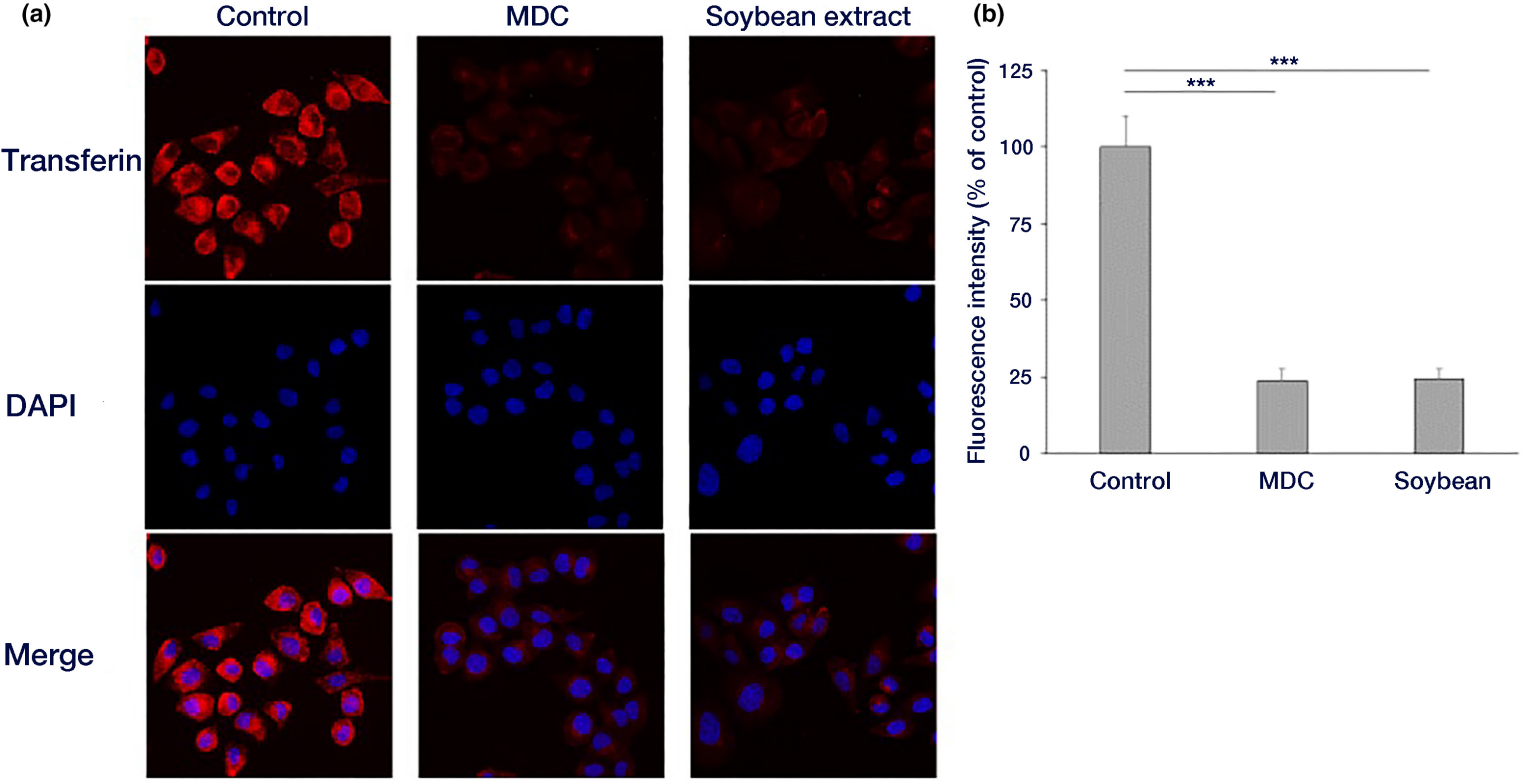
Effect of soybean hot water extract on transferrin endocytosis. (a) HeLa cells were incubated with a sample‐containing medium and kept on ice for 10 min. Thereafter, 25 μg/mL Texas Red‐conjugated transferrin was added, and transferrin was incorporated into the cells at 37°C for 10 min. After incubation, cells were fixed and observed under a fluorescence microscope. (b) The fluorescence intensity for each cell in (a) was quantified (Mean ± standard deviation of 30 cells each) (***p < .001).

The cellular uptake of transferrin was observed through fluorescence microscopy. The impact of hydrothermal soybean extract on clathrin‐dependent endocytosis was then examined. Transferrin uptake was inhibited in cells treated with hydrothermal soybean extract (Figure 7a,b), similar to that of MDC, an inhibitor of clathrin‐dependent endocytosis, indicating that the hydrothermal soybean extract affected clathrin‐dependent endocytosis.

### 3.8 Impact of soy hydrothermal extract on CTxB uptake

**FIGURE 8:**
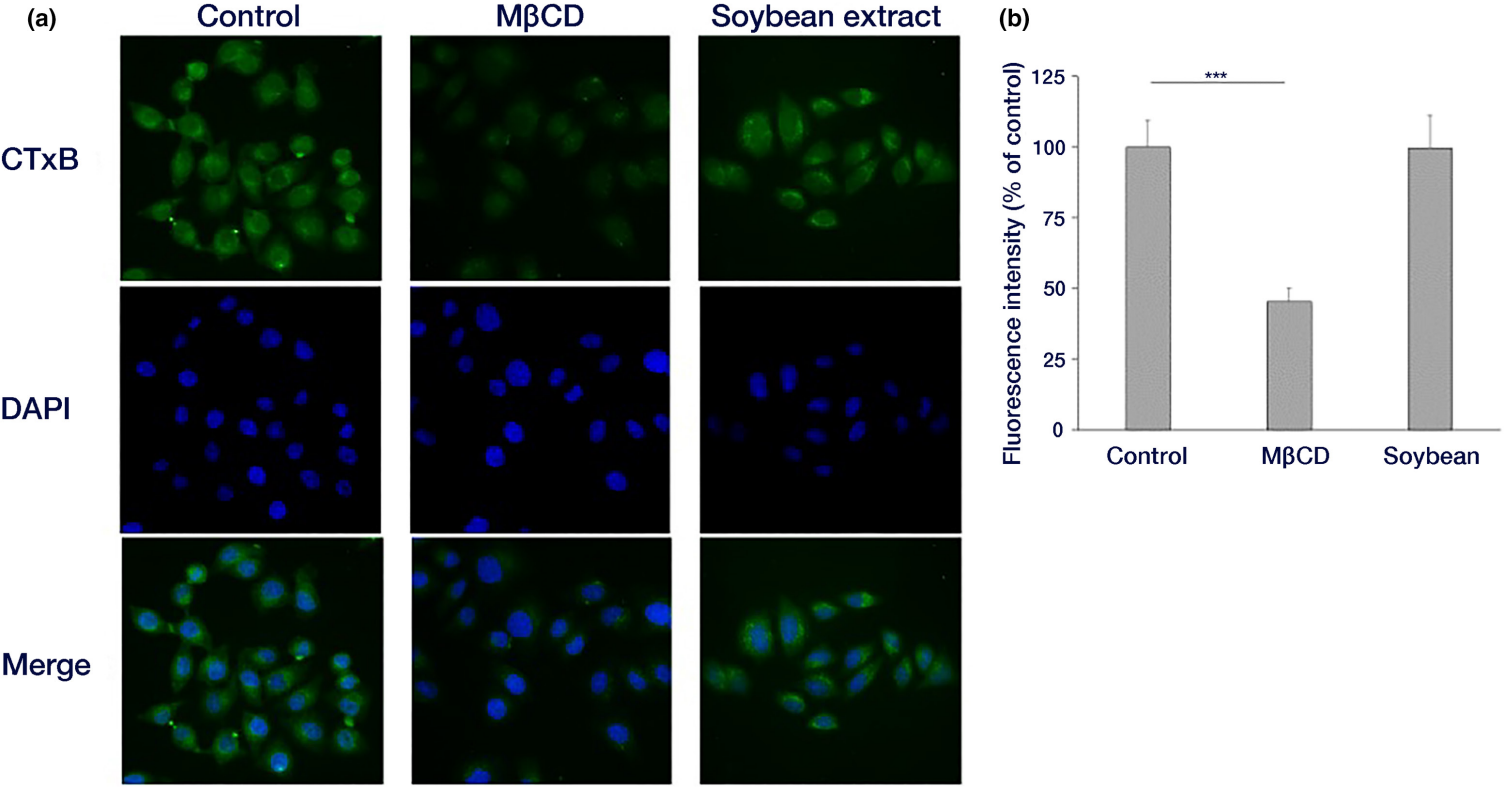
Effect of soybean hot water extract on CTxB endocytosis. (a) HeLa cells were incubated with a sample‐containing medium for 30 min at 37°C. Subsequently, 1 μg/mL Alexa Fluor 488‐conjugated CTxB was added, and choleratoxin was taken up into the cells for 5 min at 37°C. After incubation, cells were fixed and observed under a fluorescence microscope. (b) The fluorescence intensity for each cell in (a) was quantified (Mean ± standard deviation of 30 cells each) (***p < .001).

Next, we examined the influence of hydrothermal soybean extract on caveolae‐dependent endocytosis using CTxB. We observed the cellular uptake through fluorescence microscopy and conducted image analysis. The results showed no discernible disparity in CTxB uptake between control cells and those treated with hydrothermal soybean extract, although CTxB uptake was inhibited by MβCD (Figure 8a,b). This observation indicated that the soybean hydrothermal extract exerted no influence on caveolae‐dependent endocytosis.

## 4 DISCUSSION

We investigated the anti‐influenza virus effect of hydrothermal soybean extract in MDCK and HeLa cells (Figure 1). Daidzein is the only soybean extract known to be involved in the antiviral effect in both cells (Horio et al., 2020, 2023). However, daidzein inhibits the proliferative phase of the virus but not the invasive phase (Horio et al., 2020), and soybean extract inhibits the invasive phase of the virus (Figure 2); therefore, the presence of inhibitors other than daidzein is predicted. It is necessary to examine inhibitor interactions considering the total antiviral activity of soybean extracts. However, the active ingredients in soybean extracts are poorly understood, and there are no reports of interactions between soybean extracts and antiviral drugs. Yagai et al. (2024) showed synergistic antiviral effects of *Euglena* extract and quercetin, and we believe it is important to study interactions among active ingredients and interactions between active ingredients and antiviral drugs in the future. In the future, we plan to study the interactions between soybean extracts and antiviral drugs and the interactions between the active ingredients of soybeans.

In this study, hydrothermal soybean extracts strongly inhibited virus adsorption and the invasion of influenza virus infection (Figure 2). In the early stages, including virus adsorption and entry of viral infection, six processes occur: adsorption to host cells (I), uptake via endocytosis (II), endosome transport (III), viral and cell membrane fusion (IV), viral particle deshelling (V), and transport of the viral genome to the nucleus (VI) (Edinger et al., 2014). Inhibition of influenza virus adsorption by host cells through poly(ethylene glycol)ylated oleanolic acid (Zhou et al., 2022) was not observed in soybean extract. We scrutinized the effects of soybean hydrothermal extract on each of these processes. The extract showed no inhibitory effect on viral adsorption to host cells or membrane fusion (Figure 3b). Although Figure 3b indicates a slight reduction in virus adsorption with the application of soybean extract, the non‐concentration‐dependent nature of this decrease suggests that it is not the primary inhibitory mechanism of soybean extract. The inhibition of HA by epigallocatechin gallate (EGCG) is caused by disruption of the lipid bilayer rather than a direct interaction between EGCG and viral HA (Kim et al., 2013). However, temperature‐dependent virus uptake into host cells was inhibited by the soybean extract in a concentration‐dependent manner (Figure 3a). Soybean extract inhibited clathrin‐dependent endocytosis (Figure 4) but not caveolae‐dependent endocytosis (Figure 5). This may be due to the direct inhibition of clathrin‐dependent endocytosis rather than disruption of the bilayer membrane. Thus, we inferred that endocytosis is the key mechanism by which soybean extract inhibits viral infection. Subsequently, we stained influenza virus NPs to observe their effects on viral uptake and confirmed that soybean hydrothermal extract hinders viral uptake into cells, that is, endocytosis (Figure 6).

Endocytosis, involving the uptake of extracellular substances by the cell, encompasses the formation of vesicles that envelop substances by either sinking or protruding into a portion of the cell membrane, transporting the substances into the cytoplasm. Endocytosis is classified according to the vesicle components, formation mechanisms, and several pathways such as clathrin‐dependent endocytosis, caveolae‐dependent endocytosis, and macropinocytosis (Edinger et al., 2014; Lakadamyali et al., 2004). Among endocytic pathways, clathrin‐dependent endocytosis, wherein clathrin in the cytoplasm forms clathrin‐coated vesicles to facilitate substance uptake, has been extensively studied (McMahon & Boucrot, 2011). In our investigation of multiple endocytic pathways, we initially focused on clathrin‐dependent endocytosis, as caveolae‐dependent endocytosis is not utilized for influenza virus uptake into HeLa cells (Sieczkarski & Whittaker, 2002). We examined the effects on clathrin‐dependent endocytosis, and the inhibition of hydrothermal soybean extract was similar to that of MDC (Guo et al., 2015), an inhibitor of clathrin‐dependent endocytosis. This suggests that the hydrothermal soybean extract selectively impedes the clathrin‐dependent endocytosis pathway (Figure 7). We also examined the effects on caveolae‐dependent endocytosis, leaving the caveolae‐dependent endocytosis pathway unaffected (Figure 8). These results suggest that hot‐water soybean extract hinders viral entry into cells by specifically blocking clathrin‐dependent endocytosis. In the context of phytoestrogen functions, genistein inhibits caveolae‐dependent endocytosis but not the entry of the influenza virus (Sieczkarski & Whittaker, 2002). Resveratrol inhibits the viral RNA polymerase of SARS‐CoV by increasing intracellular levels of zinc (Te Velthuis et al., 2010). Notably, although this series of events is similar to the inhibition of viral entry by 8‐prenylnaringenin (Hanada et al., 2022), this is the first report of inhibition of clathrin‐dependent endocytosis by a food component through the suppression of entry of the influenza virus.

In the case of clathrin‐dependent endocytosis, over 40 proteins have been reported to be involved in uninterrupted progression (Edinger et al., 2014; Taylor et al., 2011), with certain proteins exhibiting interactions with multiple partners, including FCHO, AP2, clathrin, dynamin, auxilin, and GAK. Identification of the active ingredients in soybean extracts is expected to contribute to elucidating the detailed inhibition mechanism of clathrin‐dependent endocytosis. Some limitations in using soybean extracts to investigate the detailed inhibition mechanism of clathrin‐dependent endocytosis and its interaction with related proteins should be addressed. This study involved in vitro experiments, and there are no indications regarding efficacy in animals or humans. If it can be applied to animals, this may extend to the development of defense methods for viruses that use endocytosis in the progression of infectious diseases. In cancer, it may apply to the suppression of osimertinib resistance in EGFR‐mutated sensitive cancer cells.

In the future, we aim to identify the specific inhibitors of clathrin‐dependent endocytosis within soybean extracts and examine their effects on the proteins and factors essential for the continuous progression of clathrin‐dependent endocytosis.

## 5 CONCLUSION

In summary, the strong anti‐influenza effect of soybean extract was demonstrated during the viral entry phase, and the inhibition mechanism included suppression of entry rather than adsorption of the virus into the cells. The inhibition was not caused by inhibition of endosomal acidification or fusion of the viral membrane with the plasma membrane but by inhibition of clathrin‐dependent endocytosis. The next challenge is searching for soybean extract components that inhibit clathrin‐dependent endocytosis.

## AUTHOR CONTRIBUTIONS

**Natsumi Sakata:** Data curation (lead); formal analysis (lead); investigation (lead). **Yuka Horio:** Data curation (equal); formal analysis (equal); investigation (equal); supervision (equal). **Ryoichi Yamaji:** Supervision (lead). **Yuji Isegawa:** Conceptualization (lead); funding acquisition (lead); project administration (lead); supervision (equal); writing – original draft (lead); writing – review and editing (lead).

## CONFLICT OF INTEREST STATEMENT

The authors declare no conflicts of interest.

## ETHICS STATEMENT

This study does not involve any animal or human testing.

## Data Availability

Data will be made available upon request.
